# Role of autophagy in tumor response to radiation: Implications for improving radiotherapy

**DOI:** 10.3389/fonc.2022.957373

**Published:** 2022-09-12

**Authors:** Amrita Roy, Soumen Bera, Luciano Saso, Bilikere S. Dwarakanath

**Affiliations:** ^1^ Department of Biotechnology, Indian Academy Degree College (Autonomous), Bengaluru, Karnataka, India; ^2^ B. S. Abdur Rahman Crescent Institute of Science and Technology, Chennai, India; ^3^ Department of Pathology, University of Illinois at Chicago, Chicago, IL, United States; ^4^ Department of Physiology and Pharmacology "Vittorio Erspamer", Sapienza University, Rome, Italy; ^5^ Central Research Facility, Sri Ramachandra Institute of Higher Education and Research Institute, Chennai, India

**Keywords:** autophagy, radiotherapy, cell death, tumor microenvironment, DNA damage repair

## Abstract

Autophagy is an evolutionary conserved, lysosome-involved cellular process that facilitates the recycling of damaged macromolecules, cellular structures, and organelles, thereby generating precursors for macromolecular biosynthesis through the salvage pathway. It plays an important role in mediating biological responses toward various stress, including those caused by ionizing radiation at the cellular, tissue, and systemic levels thereby implying an instrumental role in shaping the tumor responses to radiotherapy. While a successful execution of autophagy appears to facilitate cell survival, abortive or interruptions in the completion of autophagy drive cell death in a context-dependent manner. Pre-clinical studies establishing its ubiquitous role in cells and tissues, and the systemic response to focal irradiation of tumors have prompted the initiation of clinical trials using pharmacologic modifiers of autophagy for enhancing the efficacy of radiotherapy. However, the outcome from the Phase I/II trials in many human malignancies has so far been equivocal. Such observations have not only precluded the advancement of these autophagy modifiers in the Phase III trial but have also raised concerns regarding their introduction as an adjuvant to radiotherapy. This warrants a thorough understanding of the biology of the cancer cells, including its spatio-temporal context, as well as its microenvironment all of which might be the crucial factors that determine the success of an autophagy modifier as an anticancer agent. This review captures the current understanding of the interplay between radiation induced autophagy and the biological responses to radiation damage as well as provides insight into the potentials and limitations of targeting autophagy for improving the radiotherapy of tumors.

## 1 Introduction

Since its discovery in 1898, ionizing radiation exposure has been used to eradicate cancer cells by inflicting DNA damage (1). Present-day radiation therapy (RT), along with chemotherapy, immunotherapy, hormone therapy, and surgery has established itself as one of the principal therapeutic modalities employed for the treatment of cancer. It is often combined with other therapeutic modalities like surgery, chemotherapy, and immunotherapy as this approach has been found to provide better tumor control in many human malignancies (2–4). The RT regimen—comprising of the total dose and the fractionation schedule, including dose per fraction— is designed based on several factors that include the histopathological type and anatomical location of the malignancy (5, 6), while the genetic profile (viz. status of p53, VEGF, EGF, etc.) and the physiological status (7) play a crucial role in determining the outcome of RT. The biological responses of RT at the cellular, tissue, and systemic levels depend on the type and quality of radiation, the nature of macromolecular lesions induced as well as the molecular responses elicited, which are a set of interconnected signaling pathways regulated by the genomic and proteomic status of cells—all these cumulatively drive the irradiated cells to either towards death or survival (8).

Autophagy, meaning “self-eating” in Greek, can be defined as the cellular phenomenon through which senescent, damaged, or malfunctioning biomolecules and organelles are targeted for lysosomal degradation. It is an evolutionarily conserved cellular process that is activated in response to a multitude of intrinsic and extrinsic stressors like depletion of nutrients or growth factors, infection, or hypoxia (9, 10). Under such conditions, autophagy acts predominantly as a survival response by eliminating the damaged organelles or toxic aggregates whose presence otherwise would have triggered the apoptotic response. Simultaneously, lysosomal degradation of the redundant cellular components generates valuable raw materials and nutrients that can be reused to reconstruct important biomolecules.

Though initially conceived as a pathway employed to dispose of damaged or degraded cellular organelles and biomolecules, autophagy have emerged as one of the key mechanisms involved in the modulation of several cellular processes like metabolic homeostasis (11), apoptosis (12), and the development and differentiation (9, 13). Deregulation of the autophagic process is observed in numerous diseases like neurodegenerative disorders and cancer. As such targeting autophagy, besides other response like senescence and various death pathways has recently gained interest as an approach to improve the efficacy of anticancer therapies (14). The role of autophagy in the radiation response at the cellular and tissue levels is emerging wherein the facilitation of survival or progression to death has been observed, besides contributing to tissue responses as well. This review discusses the interplay between autophagy and tumor responses to ionizing radiation and emphasizes on the clinical responses of the cancer cells towards a combination therapy of radiation with autophagy modulators.

## 2 Radiation response of tumors

Radiotherapy (RT) is one of the major armamentariums of cancer therapy that employs either photon based low LET (Linear Transfer of Energy—i.e. the amount of energy that is transferred by the radiation beam per unit distance it travels through the biological matter) radiation like X-rays and gamma-ray photons or/and high LET particles like proton, carbon ion, etc. Several forms of external beam irradiation and internally delivered radiation are currently employed depending on the nature of the malignancy and anatomical location of the tumor (15, 16). Despite significant advancements in RT technology providing a differential dose distribution between the tumor and the adjoining normal tissues (or organs at risk; OAR), acute and/or late toxicity in the non-target normal tissues or organs do compromises the clinical efficacy of radiotherapy (17).

### 2.1 Molecular and cellular responses of cancer cells towards IR

**Figure 1:**
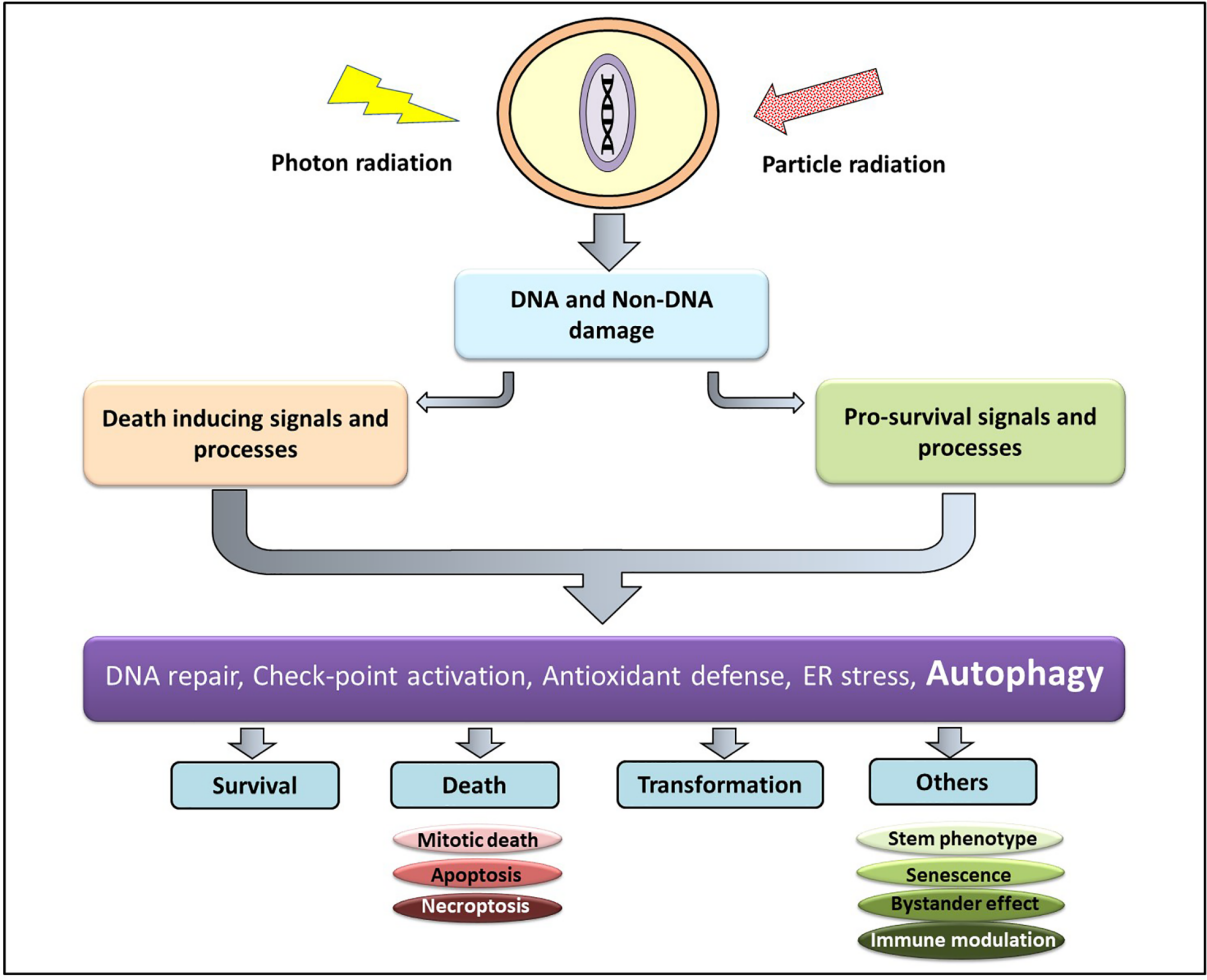
Cellular responses to radiation damage. Radiation induced macromolecular damage (DNA and non-DNA) activates pro-survival and death processes regulated by several proteins (ATM, p53, ATF6, Atg, NFκβ etc) whose level and activity are regulated majorly by post-translational modifications (which can be targeted for therapeutic benefit). This results in survival, death, and transformation of the irradiated cells, besides other responses.

At the cellular level the effect of Ionizing radiation (IR) can be both direct and indirect. The direct interaction of radiation with the macromolecules (particularly DNA) and their subsequent damage is referred to as the direct effect, while the indirect effect is brought about by the interaction between the macromolecules with the highly reactive molecular species generated due to radiation (18). Low LET radiations (X-rays and gamma-ray photons) causes damage majorly through the indirect effect, thereby are subject to the environmental conditions of the cell (particularly the oxygen level), while damages induction by high LET or particle radiations (protons, carbon, α particles, and neutron)are determined mainly by the track structures and are influenced little by the environment (19). The short-lived and highly reactive oxygen and nitrogen species generated from the ionization of cellular water react with macromolecules in the vicinity (DNA, RNA, lipids, and proteins) to generate DNA strand breaks (both single and double), lipid peroxides, and oxidized proteins. Accumulating evidences also suggest that complex DNA damage in the form of a cluster of damages comprising DNA strand breaks and a variety of non-break types of DNA damage viz. base damages play a critical role in determining the cellular and tissue responses to both low and high LET IR (20). Thus, DNA damage (particularly DNA double-strand breaks) and non-DNA damages in the form of membrane damage and imbalances in the cellular metabolism collectively determine the fate of an irradiated cell. The DNA damage response (DDR) comprises of the hierarchically regulated pathways of DNA repair, pro-survival signaling, perturbations in cell cycle progression, various cell death processes (interphase as well as mitotic), alterations in antioxidant and metabolic pathways, induction of senescence, autophagy, stem cell phenotype, bystander responses, and immune signals (**Figure 1**). A spatiotemporal competition between these pathways determine the fate of the irradiated tumor and non-malignant cells that translates into the therapeutic benefit (21) of radiotherapy. Additionally, the tumor microenvironment (consisting of stromal cells, immune cells, endothelial cells, and adipocytes), cancer stem cells, and the immunological responses of the host also contribute to the radiosensitivity of the cancer cell in determining the efficacy of radiotherapy.

The DNA damage response pathway is a multi-gene-multistep process and is greatly influenced by the post-translational modifications of several regulatory proteins that sense, transduce, and orchestrates (effectors) the dynamic interplay between, DNA repair, cell cycle arrest, mitotic death, interphase death, autophagy and senescence (22) thereby determines the survival or death of the irradiated cells (23). Ataxia-telangiectasia mutated (ATM) and the MRN complex (Mre11-Rad50-Nbs1) are two important members among the sensor proteins of DSB that play an important role in initiating DNA repair. They phosphorylate the histone variant, H2AX (gamma H2AX; γH2AX) creating a platform (template) for the progress of the repair and other events viz. cell cycle arrest in a p53 dependent and independent manner, involving other proteins like CHK1, CHK2, GADD45, CDK1, etc. DNA double-strand breaks (DSB) caused by IR primarily activates non-homologous end joining (NHEJ) and/or homologous recombination (HR) repair pathways in a context-dependent manner that like cell type, and proliferation status (24, 25). The DNA-dependent protein kinase (DNA-PK) and the RAD50 complex play a predominant role in the G1/early S phase cells, while the homology dependent HR requiring the RAD52 complex acts mainly in the late S/G2 phase, or the breast cancer predisposition genes BRCA1/2 complex, in the S phase of the cell cycle (26). Inhibitors of the DNA repair pathway and CHK1 inhibitors which sensitize cancer cells to ionizing radiation are currently under evaluation in different phases of clinical trials (22).

Although DNA damage response (DDR) plays the major role in the cellular responses to IR, non-DNA damage like membrane damage and damage to other organelles also contributes to the ultimate IR response. Ceramides generated from membrane damage induce apoptosis (8) that add to or synergize with the DNA damage-dependent apoptosis in determining the extent of cell death. Necroptosis, a regulated form of necrosis orchestrated by receptor interacting proteins (RIPK1 and RIPK3) and mixed lineage kinase like (LIKE) protein is also induced by IR and has recently been found to be involved in the activation of antitumor immunity (27). IR also enhances the unfolding of proteins due to the damage caused by radiation-induced ROS, leading to an unfolded protein response (UPR) in endoplasmic reticulum (ER) (28), which triggers the release of calcium stored in ER to the cytoplasm and causing the activation of ER stress mediated by UPR response (28, 29). A strong correlation exists between UPR response and autophagy, thus suggesting an association between radiation-induced ROS, ER stress, intracellular calcium level and autophagy (30). In addition to these many non-coding RNAs viz. the micro RNAs, long non-coding RNAs, and circular RNAs that regulate several DDR and other damaged molecular pathways, have also been shown to play crucial role in the cellular responses towards radiation which (31).

### 2.2 Systemic responses of cancer cells towards IR

Besides its effect on the irradiated cells or tissue, IR has also been found to affect the distant, un-irradiated cells or tissues in an organism in a manner that mimic the response of an irradiated cell or tissue. This phenomenon is known to as the non-target effects (NTE). NTE in a given tissue (or cells in a 2D or 3D cell culture) is widely referred to as the radiation-induced bystander effect or RIBE.

RIBE is often mediated by intercellular interactions (through gap junctions and associated proteins),secretory factors related to the radiation-induced damage called the “damage-associated molecular patterns (DAMP)” as well as others like exosomes that could contain a cocktail of microRNAs (32). As demonstrated by a variety of paradigms, phenomenologically, the RIBE can elicits several responses in the non-target cells (that are similar to the responses in irradiated cells) including autophagy, albeit to a lesser extent generally (20, 33) and has been linked to many hallmarks of cancer, including autophagy and enhanced radioresistance of observed in irradiated tumors (34).

Focal irradiation of the tumor can cause both cytotoxic and cytostatic effects on the irradiated cells thus leads to varying extents of local tumor control (18). Irradiation of the tumor also elicits a response at the systemic level that primarily arises from the alterations in the functional status of various components of the tumor microenvironment (TME) like endothelial cells, stromal cells, adipocytes, immune cells, etc. which could either enhance the resistance or result in the regrowth of the tumor (35). One of the interesting NTEs of tumor irradiation is the abscopal effect defined as the response to IR observed on a metastatic lesion located distally to the irradiated tumor. Focal irradiation of normal (non-malignant) tissues also elicits an abscopal effect including autophagy that involves the release of soluble factors from the irradiated tissue that contains microRNA (36, 37).

One of the important contributing factors to the systemic effects of radiation is the induction of inflammatory response initiated by damage suffered by the irradiated tissue. Increased expression of cell adhesion molecules from the endothelial cells related to vascular cell adhesion (VCAM-1 and E-selectin) as well as intercellular adhesion, (ICAM-1) that occurs as a response to irradiation, elicit inflammatory and immunological responses (38). Concurrently, HIF-1 (hypoxia-inducible factor 1) signaling, VEGF (vascular endothelial growth factor), and the chemokine CXCL12 stimulate pro-angiogenic signals, leading to angiogenesis and survival of the irradiated cells (26). Cancer-associated fibroblast also secrets modifiers of extracellular matrix and cytokines, while TGF-β signaling down-regulates the anti-tumor T cells and dendritic cells’ immunogenicity (39). Concurrently, radiation enhances the proliferative capacity and functionality of the Regulatory T cells (T_reg_) resulting in immunosuppression and tumor relapse. Interestingly, stereotactic radiotherapy (SBRT) has been suggested to increase the functionality of Tregs in the tumor microenvironment, in a TGF-β and IL33 independent manner pointing out the existence of multiple mechanisms involved in T_regs_ linked radioresistance (40). More recently, radiation-induced DNA damage (including fragments of chromatin found in the cytoplasm) well as micronuclei expressed in the daughter cells as a consequence of unrepaired or mis-repair DNA strand breaks has been shown to stimulate the cGAS-STING pathway leading to the activation of CD8+ T cells thereby enhancing the antitumor immunity and enhanced tumor response (41, 42). Interestingly, this pathway is negatively regulated as autophagy-deficient cells secrete higher amounts of IFNγ that can be suppressed with the knockdown of cGAS or STING (35).

Irradiation of the tumor is also known to induce the generation of the cancer stem cells (CSCs) which are relatively radio-resistant compared to the bulk of the tumor cells and responsible for increased tumor resistance to RT (43). Therefore, targeting CSCs or suppressing the induction of CSC has been considered to be a promising approach for improving RT. Several mechanisms underlie the radioresistance of CSC that include high anti-oxidant capacity, efficient DNA damage repair, reprogramming of metabolism, and induction of EMT as well as developmental signaling (43). Awakening of the quiescent CSCs following RT has been recently shown to result in tumor relapse and metastasis of oral cancers (44). More recently, the persistence of induced senescence has been shown to result in the development of CSC leading to therapeutic resistance (45).

## 3 Autophagy: Initiation, progression, and execution; micro, mini and macro-autophagy; mitophagy

The term Autophagy was coined by Christian de Duve in the early 60s (10) to describe the lysosome mediated degradation of redundant cellular organelles and since then autophagy has emerged predominantly as a survival response in the eukaryotic system, triggered in response to a hoard of intrinsic and extrinsic stressors like nutritional deprivation, oxidative or radiological stress. Studies conducted in the last 30 years have identified three major classes of autophagy occurring in the eukaryotic system, namely i) microautophagy, ii) chaperone-mediated autophagy, and iii) macroautophagy.

### 3.1 Microautophagy

**Figure 2:**
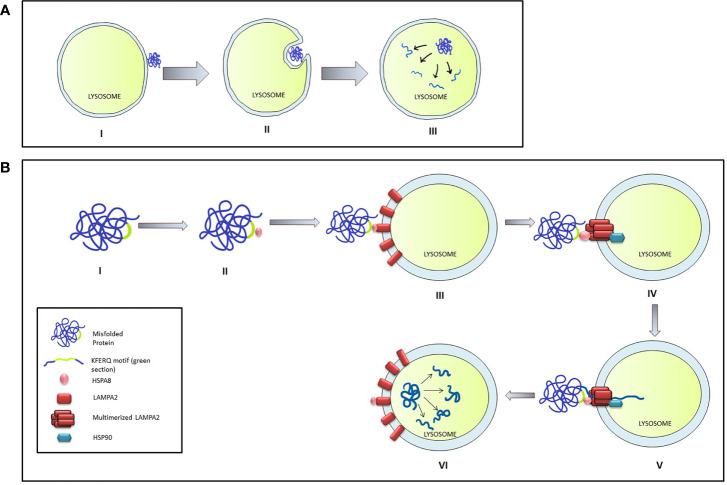
Micro-autophagy and Chaperone mediated autophagy in mammalian cells. **(A)** The stages of Micro-autophagy. I: Unfolded or damaged proteins present near the lysosomal surface triggers micro-autophagy. II: Invagination of lysosomal membrane into a cup shaped depression engulfs and degrades the damaged protein. III: Release of the cargo in the lysosome leads to its degradation. **(B)** Chaperone mediated autophagy (CMA): I & II: HSPA8 binds to the mis-folded protein by interacting through the KFERQ motif. III & IV: HSPA8 delivers the cargo on the lysosomal membrane by interacting with LAMPA2. Multimerization of LAMPA2, also mediated through HSPA2, creates a channel which is stabilized by interaction with HSP90. V & VI: HSPA8 mediates the unfolding of the protein and its translocation to the lysosomal matrix, where the cargo is degraded.

Microautophagy is a local process that occurs on the surface of the lysosome. During this process, the lysosomal membrane invaginates forming a cup-shaped depression that eventually engulfs a damaged protein or organelle and release the cargo is within the lysosomal matrix for degradation (46). Although an entire organelle can be engulfed by the microautophagy process, the uptake of cargo is essentially limited by the range of the lysosomal outer membrane (**Figure 2A**).

### 3.2 Chaperone-mediated Autophagy

The chaperone-mediated autophagy (CMA) specifically targets the targets proteins bearing the KFERQ pentapeptide motif (47) which encompass nearly 30% of all cytosolic proteins like the glycolytic enzymes, proteasomal subunit proteins, several transcription factors, and their inhibitors, calcium, and lipid-binding proteins, and proteins involved in vesicular transport. CMA is induced by stressors like oxidative stress, prolonged nutritional deprivation, and several protein-degrading toxins. During the process, the target protein is identified and delivered on the lysosomal surface through the interaction between HSP8 (heat shock protein 8) and the KFERQ motif of the target protein. Presence of target protein initiates the aggregation of LAMP2A lysosomal-associated membrane protein 2A) on the membrane surface which is stabilized by interaction with HSP90 on the luminal side of the lysosomal membrane. HASP8 unfolds target protein and delivers it through the translocation channel formed by the LAMP2A aggregate into the lysosomal lumen (46) (**Figure 2B**).

### 3.3 Macroautophagy

Macroautophagy is the most common and hence the most widely studied mechanism among the autophagy processes. Unlike the microautophagy and the CMA process, macroautophagy is initiated away from the lysosomal membrane in a specific cytosolic location. In yeast, this initiation site is known as the Phagophore Assembly Site or PAS (10, 48), although the mammalian counterpart of the PAS is yet to be established. However, during starvation-induced autophagy in the mammalian system, a certain subdomain of the ER known as the “omegasome” serve as the site for the initiation process (48). Once triggered, autophagy proceeds through four stages— initiation or nucleation, elongation, maturation, and culminates in the fusion of the autophagosome and the lysosome (49). The entire process is orchestrated by a family of conserved proteins known as the autophagy-related proteins or the Atg proteins (50).

In mammalian cells, the initiation complex is made up of either ULK1 or 2 (Unc-51 like kinase family), ATG13, and RB1CC1 (RB1 inducible Coiled-coil 1, also known as FIP200) proteins. The ULK1/2-ATG13-RB1CC1 complex is highly stable and exists within the cell even in absence of any stressors. The complex remains bound to the mTORC1 complex which phosphorylates and maintain the complex in a dormant state. However, prolonged nutrient stress dissociates the ULK1/2-ATG13-RB1CC1 complex from the mTORC1. This leads to the dephosphorylation and subsequent activation of the former and initiates the “nucleation” process (51, 52).

In both yeast and mammalian systems PIP3 generated by a novel Atg14 containing class III PI3K complex plays a crucial role in the nucleation process (46). Subsequently, PI3K forms a complex with the Beclin1 and UVRAG—an association that is crucial in the induction of macroautophagy. Several regulatory proteins are known to interact with the PI3K-Beclin1-UVRAG complex, thereby regulate the macroautophagy process. For example, Bcl2 or Rubicon is known to prevent the Beclin1 from interacting with the PI3K (53, 54) or with the PI3K-UVRAG complex (55, 56) and thereby suppress autophagy. Similarly, AMBRA1 and SH3BLG1 positively regulate the PI3K system by directly (*via* AMBRA1) (57) or indirectly (*via* UVRAG) (58) interacting with Beclin1.

#### 3.3.1 The elongation

The nucleation of the autophagy process leads to the formation of an isolated membrane structure that elongates into a cup-like phagophore which expands and after encircling the damaged cellular components forms a double membrane sphere known as the autophagosome. The expansion of the membrane structure is mediated by a couple of conjugation systems recognized as UBL (Ubiquitine-like) complexes such as Atg5, Atg12, and Atg16 or the Atg8/LC3 system. A dimer of Atg12-Atg5-Atg16 complex (formed by the covalent linking of Atg16 to Atg5-Atg12 complex) is required for the expansion of the membrane system (59).

**Figure 3:**
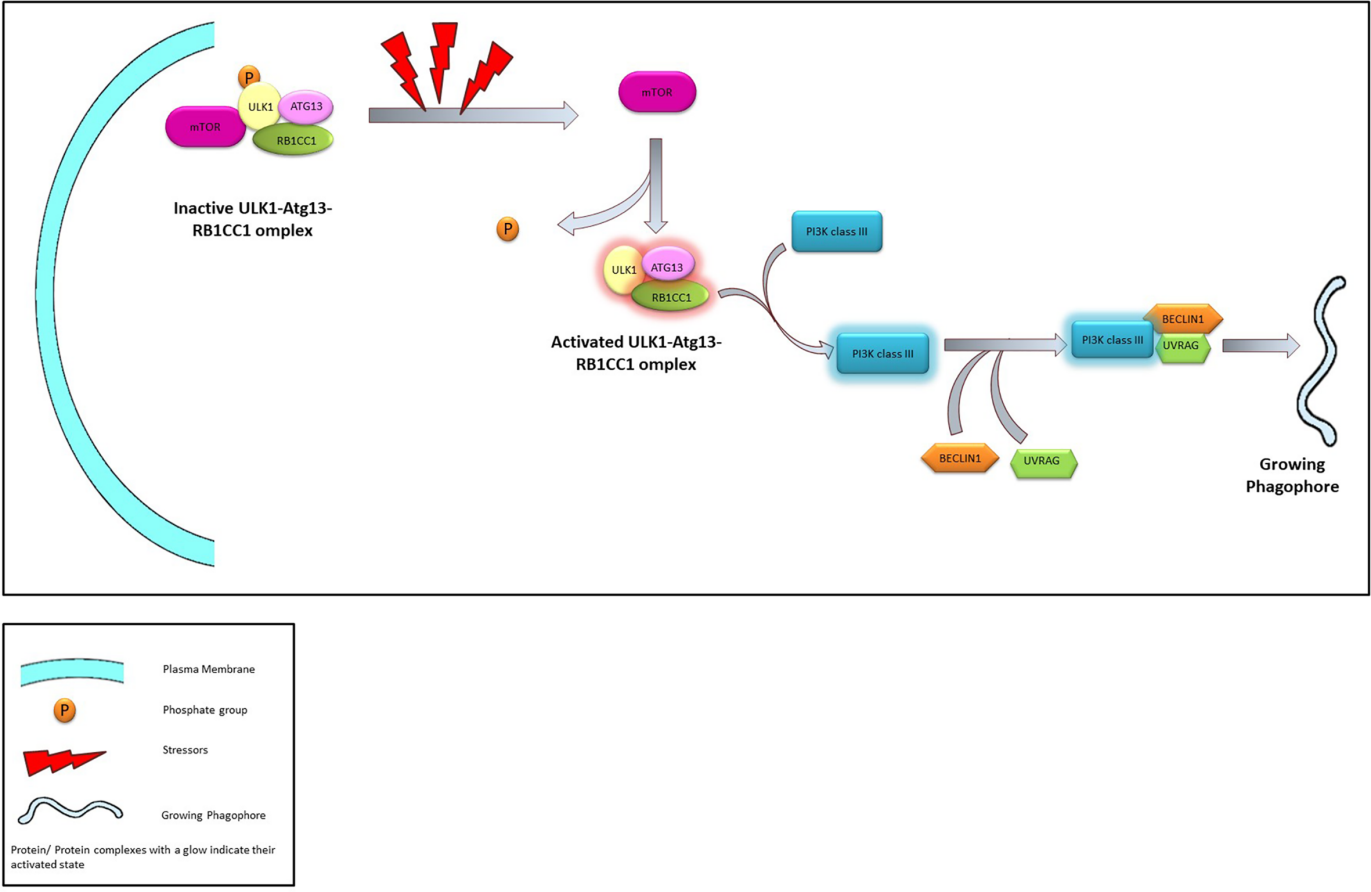
The Process of Nucleation. Under normal conditions ULK1-Atg13-RB1CC1 is maintained in an inactivated state through phosphorylation promoted by interaction with mTOR. In presence of stressors ULK1-Atg13-RB1CC1 complex is activated following dissociation from mTOR and dephosphorylation. ULK1 complex activates class III PI3K which in turn associates with Beclin1 and UVRAG leading to the initiation of phagophore.

The multimeric Atg12-Atg5-Atg16 complex, once formed, mediates the formation of the Atg8/LC3 system which is also essential for the elongation of the phagophore. Atg8 is initially cleaved by Atg4, exposing a Gly residue at its C terminal end, followed by activation by the sequential interaction with Atg3 and Atg7. The activation of Atg4 is stringently controlled by the phosphorylation by ULK1 complex (60) or by the ROS level of the cell (61). The exposure of the Gly residue is a critical step in the activation process of Atg8 as this residue is required to link the Atg8 complex with the phosphatidylethanolamine moieties of the growing phagophore membrane. This interaction is believed to be mediated by the Atg12-Atg5-Atg16 dimers (62, 63) (**Figure 3**).

**Figure 4:**
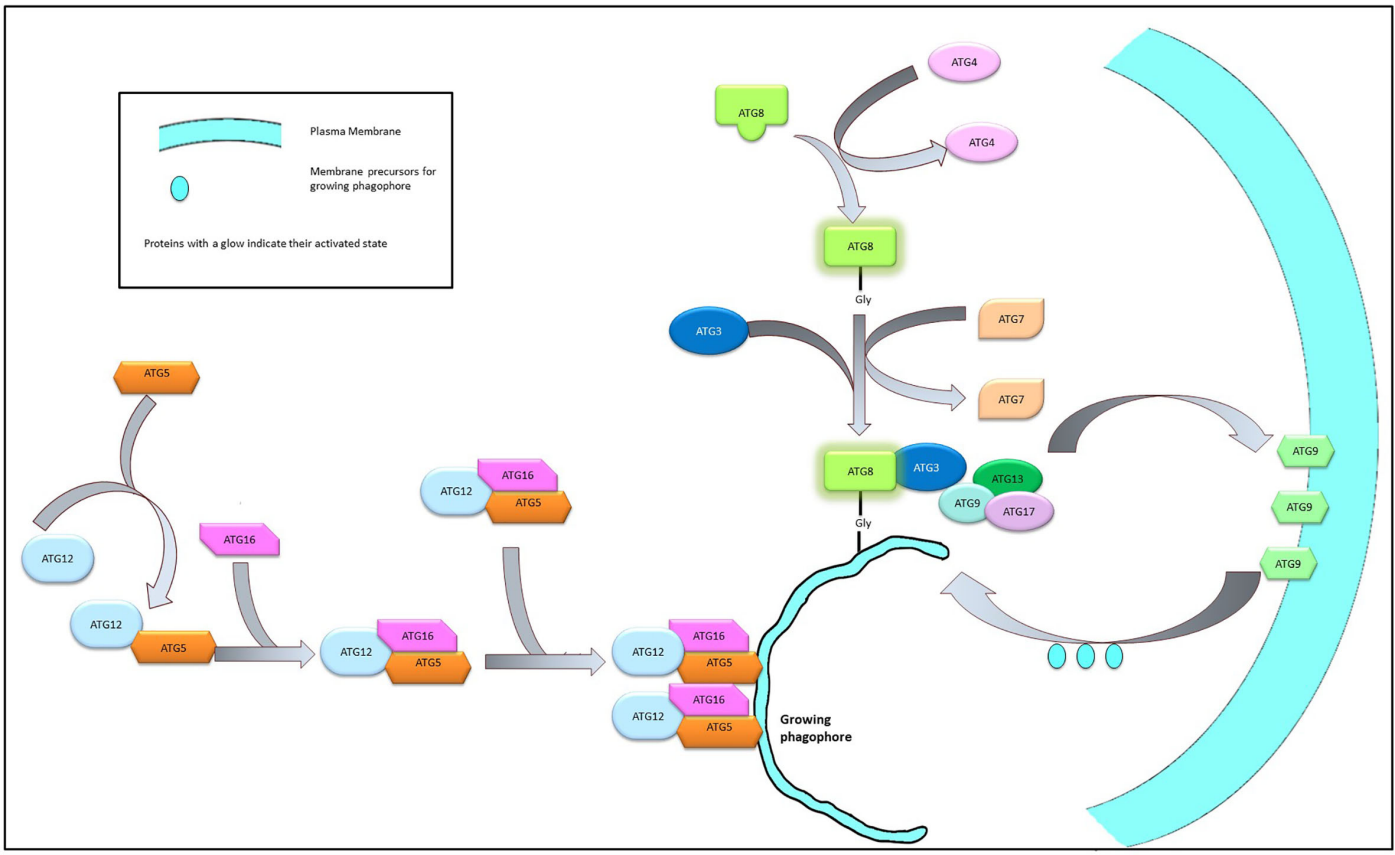
Elongation of phagophore membrane. Dimerization of the Atg12-Atg5-Atg16 complex on the surface of the growing phagophore membrane promote the recruitment of Atg8 and Atg9 complex on the growing membrane. Atg9 imports membrane components from the neighboring bio-membranes to the growing phagophore to facilitate elongation.

The membrane components essential for the elongation of the autophagosome is usually sequestered from the peripheral membrane systems—a process mediated by Atg9. Under normal physiological conditions Atg9 resides in the trans-Golgi and endosomal region. However, during nutritional stress, Atg9 reportedly migrates to the nucleation sit following an ULK1-PI3K signaling axis and shuttles between the growing phagophore and the peripheral biomembranes (64, 65). The interaction between Atg9 and Atg17 is required for the successful recruitment of the Atg9 on the autophagosome and this interaction is mediated by Atg1 complex (66). On the surface of the developing autophagosome, ATG9 is stabilized by the direct physical interaction with LC3 through specific docking domains (Ubiquitin-interacting motifs in ATG9 and UIM docking site on LC3) (67) (**Figure 4**).

The elongation/curvature of the growing autophagosome is a direct function of the Atg14 dependent class III PI3K activity. The C terminal domain of the Atg4 bears a BATS (Barkor/Atg14 autophagosome-targeting sequence) domain that facilitates its interaction with the PI3P in the lipid bilayer of the autophagosome. PI3P is responsible for a higher degree of membrane curvature (68) and it can be surmised that Atg14 acts as an indicator for membrane curvature of a budding autophagosome.

#### 3.3.2 Maturation and fusion of the autophagosome with the lysosome

In the final steps, the developing phagophore expand and close its double-membrane structure to create the autophagosome which undergoes “maturation” before its fusion with the lysosome. The maturation process is characterized by the gradual removal of the membrane-bound Atg proteins associated with the nucleation and elongation steps and the simultaneous incorporation of proteins belonging to the SNARE machinery like the VAM7, VAM9, syntaxin17, and SNAP29 (69, 70), proteins that are considered integral for membrane fusion. On completion of the maturation process, the autophagosome travels to the lysosome assisted by the microtubule system (71) and fused with the lysosome to form the phagosome.

### 3.4 Mitophagy

The name mitophagy was coined by Lemasters to describe autophagic machinery that selectively degrades mitochondria (72). Based upon the molecular machinery, mitophagy can be either PINK1/Perkin mediated, or receptor mediated both of which proceeds through initiation, priming of the damaged organelle, formation of autophagosome which fuses with the lysosome.

#### 3.4.1 The PINK1/Perkin mediated mitophagy

PINK1 is a Ser/Thr kinase whose localization on the mitochondrial membrane varies according to the changes in the membrane potential of the organelle (ΔΨ_m_). Under normal physiological conditions, PINK1 is localized in the inner mitochondrial membrane. However, disruption of ΔΨ_m_ (73, 74) or excessive accumulation of misfolded proteins (75) trigger the relocation of PINK to the outer membrane of the damaged mitochondria and its subsequent activation through autophosphorylation. Phosphorylated PINK triggers the localization of ubiquitin and Perkin on mitochondrial surface generating an “eat-me” signal that promote polyubiquitination thereby targeting the damaged mitochondrial for autophagy (76–78). In a Perkin independent pathway that involve interaction with ubiquitin chains, PINK can also mediate the accumulation of autophagy adaptors like p62, NDP52, optineurin, and ULK1 on the mitochondrial surface (78). These adaptor proteins bear LC3 domain and forms “mitophagosom” (78). Phosphorylation of opintoneurin post recruitment also feed forward the process (79).

#### 3.4.2 Receptor-mediated mitophagy

The inner and outer membrane of mitochondria houses several receptor proteins like FUNDC1, BNIP3L, FKBP8, prohibitin2, and cardiolipin (80). The localization of these receptor proteins across the inner and outer mitochondrial membrane depends on the stressor level of the cell and is essential in priming a damaged mitochondrion for elimination through different autophagic machinery. For example FKBP38, prohibitin2, and cardiolipin are known to bear LC3 domain and can promote the formation of autophagosome around a damaged mitochondria (81). Prohibitn2 also promote localization of Perkin on the mitochondrial membrane, thus interlinking different branches of mitophagy (82). Similarly translocation of cardiolipin from the inner to the outer mitochondrial membrane in response to stressors generates a potent “eat-me” signal (83) and its release from the outer mitochondrila membrane acts as a strong inducer of apoptosis (84).

These receptors are also known to mediate PINK1-Perkin independent mitophagy in high energy demanding tissues like brain (85). Under normoxic conditions, FUNDC1 phosphorylation promotes mitochondrial fusion as well as prevents mitophagy (86), whereas under hypoxic conditions, dephosphorylation of FUNDC1 by specific mitochondria-based phosphatases initiate mitochondrial fragmentation (87) and mitophagy (86, 88).

The BNIP3/NIX axis, that interlinks the mitophagy machinery with that of the general autophagy ones, is often deregulated in cancer (89). Under hypoxic conditions the BNIP3 and NIX are overexpressed [through a HIF1α mediated pathway (90)] and undergo phosphorylation. Phosphorylated BNIP3 and NIX interacts with LC3 (91, 92) and channelize the organelle for macroautophagy based elimination. BNIP3 also stabilizes that the PINK1-Perkin machinery (93), thereby not only links mitophagy with a major macroautophagy machinery, they effectively target the damaged mitochondrion for degradation and thereby suppress the production of excess amount of mtROS under hypoxic conditions (94, 95).

In addition to mitophagy several forms of autophagy have been identified dedicated to the selective elimination of specific challenges like pathogens (xenophagy), protein aggregates (aggrephagy), or damaged ER (reticulophagy). Protein aggregates—formed by the aberrant interaction of misfolded proteins— are eliminated through receptor-mediated autophagic machinery (96). Simultaneously, excessive accumulation of misfolded proteins in the ER lumen trigger reticulophagy or ER-phagy. The amelioration of ER stress is known to have significant survival implications. Moreover, autophagy processes like ferritinophagy (the receptor-mediated lysosomal degradation of ferritin that takes place under iron deprivation) (97), glycophagy (degradation of glycogen molecule by lysosomal α-glucosidase) (98), or lipophagy (lysosomal degradation of lipid droplets and lipoproteins) (99) are crucial for nutrient homeostasis and cell survival.

## 4 Influence of autophagy on radiation response of tumors

Radiotherapy although being a frontline approach for cancer treatment, often meets with failures. This is due to the radio-resistance that a growing tumor acquires through deregulation of stress responses, like the DNA damage and repair mechanisms that promote autophagy and leads to nutrient recycling. In addition to the damaged proteins, various intermediate molecules and their complexes and damaged organelles like mitochondria and micronuclei do serve as cargo for autophagy (49). Recent studies have bestowed both pro and anti survival nature to the autophagic pathways and cancer cells are known to exploit this dual nature of autophagic pathways to survive in a metabolically challenged microenvironment, to escape the host-immune responses, to evade apoptosis, and to metastasize (100, 101).

### 4.1 Effect of autophagy on IR induced DNA damage and repair

Irradiation of tumor cells ionizing radiation initiates a series of events ranging from DNA damage, ROS induction, cell death, and cell senescence with intricate crosstalk amongst themselves. It is widely acknowledged that DNA double-strand breaks that results in are majorly responsible for the initiation of the cell death that are instrumental in regulating local tumor growth. Along with the inducement of DNA damage the DNA damage response (DDR) acts pro-actively and seamlessly to prevent the accumulation of DNA damages (arising due to any stress). Once the DDR commences and the extent of the DNA damage is assessed, autophagy plays a pivotal role in deciding the ultimate fate of the cell.

Ataxia-telangiectasia mutated (ATM) and ATM/Rad 3-related (ATR) are cell cycle checkpoint regulators that also act as DNA damage sensors and are involved in activating the DDR pathways (102). ATM is involved in multiple cellular phenomena like cycle arrest, apoptosis, and autophagy and hence considered as a tumor suppressor protein (103). Moreover cancer cells are known to employ myriad pathways—like upregulation of miRNA18a (104) and WIP1 phosphatase (105)—to suppress ATM activity leading to induction of autophagy through deregulations of glucose metabolism and energy deprivation (106). However growing evidences also suggest that ATM is also involved in promoting chemo- and radio-resistance (107–109) to cancer cells which might in turn be the reflection of the dual nature of the autophagic processes that the protein initiates. Activation of autophagic pathway, through ATM-CHK2-BECN1 axis, is also observed in irradiated tumor cells exhibiting high level of oxidative stress.

### 4.2 Effect of autophagy on IR induced cell death

The radiosensitivity of the tumor cells are often mediated through suppression of the autophagic machinery. The nuclear translocation of Beclin1 is often observed in response to IR exposure which in turn leads to G2/M cell cycle arrest (110). ATG5-driven autophagy is also known to promotes the radio-sensitivity of prostate cancer cells under nutrient-starved or glutamine depleted conditions or with the silencing of MYC (111). Hence silencing the Belcin1 or ATG5 expression had been shown to reduce the IR sensitivity of the cancer cells. Similarly suppression of ATG7 by long non-coding RNA (lncRNA) HOTAIR—which significantly overexpresses in irradiated prostate cancer cell lines in response to irradiation—is associated with radioresistance in irradiated cells (112).

The association between IR and autophagic pathways is further emphasized by the observation that in breast cancer cells, which are inherently resistant to apoptosis, IR exposure results in enhanced autophagic phenotypes resulting in increased iron accumulation, which coupled with the subsequent ROS generation, oxidative stress, and DNA damage, can result in the induction of cell death through ferroptosis (113, 114). Consequently, in recent years combining autophagic inducers along with IR is emerging as an interesting approach to increase the radiosensitivity of the cancer cells (105, 106). In a similar approach, treatment of Non-small cell lung carcinoma (NSCLC) cells with rapamycin and histone deacetylase inhibitor was found to promote radiosensitization (115). This combination has dual effects of enhancing autophagy along with the inhibition of the DNA damage repair machinery and the effect was observed both in the cultured cells and in the tumor xenograft mice models.

However, the effect of autophagy on the survival of cancer cells are function of multiple aspects and as a result, autophagy can act as a promoter as well as an antagonist towards radio-sensitization. In fact, different cancer cell types have been found to benefit from enhancing autophagy as their survival strategy. For example, in presence of autophagy inhibitors, otherwise radio-resistant bladder cancer cells developed sensitivity towards chemotherapy (116). Similarly, inhibition of autophagy through ATG5 silencing is known to increase the IR-induced cell death in nasopharyngeal carcinoma (117). The autophagy inhibitors when combined with IR have emerged as one of the principal factor that influence the bystander and abscopal effects (as discussed in the later sections) observed after chemo and radiotherapy (118).

### 4.3 Effect of autophagy on cancer stem cells and IR response

Autophagy plays an important role in maintaining the ‘stem-ness’ of the CSCs. The majority of the tumors activate the epithelial-mesenchymal transition (EMT) program to attain the stem cell-like properties and to promote their growth, invasion, and metastasis. The autophagy-related genes, especially ATG5 play a critical role in the EMT process as indicated by a study on cervical cancer cells (119). In radio-resistant cancer cells, like pancreatic ductal adenocarcinoma cells and NSCLC stem cells, autophagy has found to be essential in promoting tumor growth and invasiveness (120) as well as maintaining the stem cell-like properties (121) of the cells. as a result autophagy inhibitors, either individually or with combination with other traditional methods of cancer therapy, have been able to block proliferation, colony and, spheroid formation (in pancreatic CSC populations) in cancer cells (122). Similarly, silencing of prominent autophagic genes like Atg5 was able to induce radio sensitivity within radio-resistant cancer stem cell populations (prostate CSCs) (111).

### 4.4 Effect of autophagy on Radiation Induced Bystander Effect (RIBE) and Abscopal Effect

IR-exposed cancer cells secrete hoard of signaling molecules in their microenvironment that modulate the biology of the neighboring non-transformed cells leading to what is now recognized as the Radiation induced Bystander Effect (RIBE). RIBE is one of the principal factor that is considered to modulate the cytotoxic effects of radiation in the irradiated tumor targets (34). Usually the bystander cells responds to the challenge by secreting a number of cytokines like IL6 (123), IL1, TNFα, IL18 (124), colony-stimulating factor 2 (CSF2)/JAK2/STAT3 (125), as well as microRNAs such as microRNA-7 (36), microRNA-7-5P (33), and ROS (126). All these molecules significantly influence the crosstalk between cancer and the neighboring non-irradiated cells that often translates in altered autophagy (33). RIBE have often been mediated by regulation of autophagy that exhibit spatial and temporal differences. The exosomal miRNAs like miR17-5p that are secreted by the irradiated cancer cells are known to induce autophagy in non-irradiated bystander cells while suppress the same within themselves (33). In irradiated HeLa cell culture, the bystander cells have been shown to exhibit enhanced autophagy, providing nutrient supplies to the nutrient-deficient cancer cells (123) while in irradiated glioma cells higher level of miR17-5p or miR273 results in pronounced antitumor effect through suppression of autophagy (127, 128). Exosomes containing miR7-5-p are known to induce autophagy in neighboring non-irradiated cells through suppression of the EGRF-Akt-mTOR axis resulting in radiation induced tissue damage (129).

Apart from systemic level, autophagy appears to be a crucial mediator of RIBE/tumor response to radiation at the organelle level. Mitophagy is often induced in irradiated cancer cells through which the mitochondria, damaged by the excess mtROS produced due to radiation, renew themselves (130). However, the dichotomous nature of autophagy is also reflected in such cases as well. In the bystander HepG2 cells, increase in the level of ROS production reflects in higher expression level of autophagic proteins LC3II/I and Beclin 1, suggesting that ROS level might be a critical determinant between the cytotoxic and cytoprotective nature of autophagy (126).

## 5 Impact of autophagy modifiers on radiation response of tumors: pre-clinical studies

Autophagy is considered as one of the very first cellular processes activated in response to radiation onslaught and although Consequently autophagy modifiers have immense importance in radiation therapy considering the dual role of autophagy in tumor formation, aggression, and metastasis (101). However, the role autophagy on cancer cells changes with the stage and progression of the tumor mass. In the initial stages of a tumor, autophagy plays a predominantly tumor suppressor role (131) while in the established tumor, where autophagy protects (cytoprotective autophagy) the cancerous cells against different stresses, helps them survive, and gain therapy-resistant phenotypes (132). Consequently, the inclusion of autophagy inducers in the treatment regimen might have a cancer-suppressing effect during the early stages of malignancy, but an autophagy inhibitor may have better radio-sensitizing efficacy in the later stages of the disease. Hence—in spite of their promises—application of the autophagy modifiers in cancer therapy, are considered to be strategically challenging and are yet to gain favor as a therapeutic modality. In this section, a broad overview of the different types of autophagy modifiers, with their reported applications in the preclinical models of cancers, has been discussed (**Table 1**) to elucidate the complex role of autophagy in determining the success of RT.

**Table 1:** Preclinical studies involving Autophagy modulators, the molecular mechanism they employ and, their effect on response to RT.

Autophagy modifier	Mechanisms reported in the study		Response to IR	Pre-clinical model	Reference
**Autophagy Induction**					
Rapamycin		mTOR inhibition, downregulation of Survivin expression; Reduced clonogenicity	Radiosensitization	Glioma cell line and mouse xenograft	(133, 134)
		mTOR inhibition, impaired DNA damage repair	Radiosensitization	Breast cancer cell line	(135)
		mTOR inhibition	Radioresistance	C57BL/6 Mice	(136)
Rapamycin + ABT-737		Apoptosis induction	Radiosensitization	Non-small cell lung carcinoma and mouse xenograft	(137)
Temsirolimus		mTOR inhibition	Radiosensitization	Renal cancer cell line	(138)
Everolimus		mTOR inhibition	Radiosensitization	Prostate cancer cell lines	(139)
M867 + Everolimus		mTOR inhibition and apoptosis inhibition	Radiosensitization	Lung cancer cells	(140)
PCI-5002		Apoptosis inhibition	Radiosensitization	Lung cancer cells and mouse xenograft	(141)
BEZ235 + PI103		PI3K/mTOR inhibition, cell cycle arrest, apoptosis induction	Radiosensitization	Prostate cancer cell lines	(142, 143)
NVP-BEZ235 + AZD6244		Inhibition of mTOR and MAP Kinase pathway	Radioresistance	Lung and glioma cell lines	(144)
Pevonedistat/MLN4924		Inhibition of NEDDylation	Radiosensitization	Liver cancer cell lines	(145, 146)
**Autophagy Inhibition**					
NVP-BEZ235 + 3MA or Chloroquine		Inhibition of PI3K/mTOR, apoptosis induction	Radiosensitization	Head and neck carcinoma and glioblastoma cells	(147)
Chloroquine		Apoptosis induction	Radiosensitization	C57BL/6 Mice	(136)
Chloroquine		Apoptosis induction	Radiosensitization	Colorectal cells	(148)
Chloroquine + Temsirolimus		Inhibition of mTOR, induction of apoptosis	Radiosensitization	Colorectal cells	(149)
Everolimus + Chloroquine		Inhibition of mTOR, induction of apoptosis	Radiosensitization	Neuroendocrine cells	(150)
Hydroxychloroquine		Apoptosis induction	Radiosensitization	Colon cancer cells	(151)
3-MA		PI3K inhibition and apoptosis induction	Radiosensitization	Esophageal cancer cells and mouse xenograft model	(152, 153)
Tunicamycin + 3-MA		ER stress induction and apoptosis	Radiosensitization	Esophageal cancer cell	(154)
Core-shell copper selenide-coated gold nanoparticles		Lysosomal alkalization, impaired DNA damage repair	Radiosensitization	Glioblastoma cells	(155)

Autophagic modulators, generating radioresistance are indicated in red highlighted box.

The autophagy inducers are usually the nutrient or ER stress inducers, or antagonists of the mTOR blocker rapamycin (and its derivatives). ER stress induction is accompanied by the downstream activation of autophagy and the appearance of autophagolysosomes. Hence an induction of the autophagic flux was observed when EC109 esophageal cancer cell line is treated tunicamycin (a ER stress inducer) (154). However, when irradiated EC109 cells were treated with tunicamycin along with 3-methyladenine (3-MA), and autophagy inhibitor, an increased apoptosis was observed in the treated cells, suggesting the involvement of autophagy in rescuing irradiated cells from apoptotic cell death (154).

The PI3K/Akt/mTOR pathway is known to suppress the ER-induced autophagy pathways and consequently mTOR—a serine/threonine kinase and an integral part of the PI3K/Akt/mTOR signaling pathway—have been targeted in many *in vitro* and *in vivo* studies to regulate the outcome of RT. Rapamycin and its derivatives everolimus, temsirolimus, deferolimus, zotarolimus, etc. are well-known TOR kinase inhibitors that have been employed in combination treatments to increase both chemo-therapeutic and radio-therapeutic efficacies. Rapamycin has been demonstrated to increase the efficacy of fractionated radiation against glioma xenograft models (133). In glioblastoma cells, rapamycin pretreatment has increased radiosensitivity with reduced expression of surviving and clonogenic potential (134). One of the major pathways involved in rapamycin-induced autophagy induction (*via* mTOR inhibition) and subsequent radio-sensitization is by impairing the DNA damage responses, specifically the homologous recombination and the non-homologous end-joining mechanisms (135). Rapamycin blocks the recruitment of BRCA1 and Rad51 to the damaged DNA thereby inhibiting the downstream pathways of homologous recombination.

Although rapamycin has been successfully used in *in vitro* cell cultures its low solubility in an aqueous system limits its application as a potential therapeutic agent. Hence its analogs, with better water solubilities, are currently used in cancer therapeutics. In renal cancer cell lines which are deficient in the VHL (von Hippel-Lindau) tumor suppressor gene, inhibition of the late-stage autophagy by small molecule inhibitor, STF-62247 or temsirolimus (the first FDA-approved mTOR inhibitor) has better radiosensitization effects than the individual treatments (138). Everolimus, another rapamycin analog, enhanced the radiosensitivity of the prostate cancer cell lines, PC3 and DU145, in a PTEN (phosphatase and tensin homolog) dependent manner (139) with PTEN-deficient PC3 cells exhibiting higher susceptibility to radiation with significant autophagy induction. Moreover, blocking apoptotic pathways in these cells had increased radiation-induced autophagic cell death.

PTEN is a tumor suppressor and metabolic regulator which has a profound role in cell division and proliferation by negatively regulating the PI3K/Akt/mTOR pathway and it is frequently mutated or inactivated in tumors. Loss of PTEN induces radioresistance in cancer cells by the downregulation of radiation-induced autophagic cell death which has been observed to be overcome by treating the cells with mTOR inhibitors. In non-small-cell lung cancer cell line HCC827, which are refractory to gefitinib, an EGFR tyrosine kinase inhibitor (TKI) possibly due to PTEN deficiency has been radiosensitized by treating with mTOR inhibitors with activation of autophagic flux (156).

Application of dual PI3K/mTOR inhibitors, BEZ235 and PI103, in combination with IR, had shown superior anticancer efficacies and enhanced radiosensitization characterized by reduced colony-formation, G2/M cell cycle arrest, increased DNA damage, apoptosis, autophagic flux in the radioresistant prostate cancer cells (128). In prostate cancer radioresistance is largely modulated by the PI3K/Akt/mTOR activation in association with an epithelial-mesenchymal transition (EMT)/cancer stem cell-like phenotype. Treating these cells with the dual inhibitor BEZ235 induced apoptotic cell death which helped to overcome the radioresistance suggesting BEZ235 to be a promising candidate for combination therapy in prostate cancers therapeutics (142). Similarly NVP-BEZ235—another a novel PI3K/mTOR inhibitor—had exhibited promising autophagy induction and enhanced radiosensitivity and apoptosis in human glioma stem cells (157) through blocking the DNA damage repairing pathway. However, when combined with temozolomide, an alkylating agent, NVP-BEZ235 has been shown to downregulate PI3K/mTOR pathways, in glioma cells (158) but in combination with AZD6244, a MAP kinase inhibitor, NVP-BEZ235 significantly reduced radio-sensitization of irradiated lung cancer and glioma cells (144). One of the reasons for the contradictory behavior of NVP-BEZ235 could be the different mechanisms through which it enhances radiosensitization and induces autophagy as suggested by Cerniglia et al. (147). Although autophagy was induced by NVP-BEZ235 in cancer cells but using the autophagy inhibitors 3MA or CQ in NVP-BEZ235-treated and IR exposed cells, had increased the level of cytotoxicity.

Emerging evidence indicates improved radiosensitization of cancer cells when combined with NEDDylation inhibitor MLN4924 (also known as Pevonedistat) with augmented autophagy induction associated with DNA damage, apoptosis, and senescence (145, 146, 159). NEDDylation (conjugation of NEDD8 moiety to protein substrates) which is tightly regulated in normal cells, targets crucial tumor suppressor proteins towards degradation and is highly active in cancer cells (160); hence, NEDDylation inhibitors are conspicuous contenders in anticancer therapeutics. Although it has been predicted that the mode of autophagy induced by Pevonedistat is protective and promotes tumor drug resistance but the inclusion of autophagy inhibitor along with it showed promising antitumor effects (161). With significant successes in the preclinical studies, Pevonedistat is currently under clinical phase I/II trials (162) even though investigational studies on it as radiosensitizer is limited.

Interestingly, induction of autophagy by modulating the apoptotic signaling cascade has been studied in some preclinical models. The lung cancer cells had turned radio-sensitive when treated with zinc ionophore PCI-5002 (141) or with apoptosis inhibitor, M867 in combination with everolimus (140). On the other hand, rapamycin along with ABT-737, an apoptosis inducer, enhances the radiotherapy response of the non-small cell lung cancer cells (NSCLC), both in *in vitro* and *in vivo* xenograft mice models with an almost 6-fold induction in the autophagic flux compared to the radiation only group (137). One reason for the synergistic effect could be the application of ABT-737 which induces apoptosis in a Bax/Bak-dependent pathway. The NSCLC cells are Bax deficient due to the overexpression of Bax inhibitor-1 protein (163), which could be compensated by the upregulation in Bax expression brought about by the rapamycin treatment, thereby enhancing the ABT-737 mediated apoptosis (164).

In a very recent approach core-shell Copper selenide coated gold nanoparticle was used to improve the response of the glioblastoma cells towards RT. The nanoparticles impaired the autophagic machinery by alkylating lysosomes leading to inactivation of the lysosomal enzymes within. Simultaneously the nanoparticles increased the ubiquitination and protosomal degradation of the DNA repair protein Rad51, thereby compromised the repair of the DNA strands damaged by irradiation. The cumulative effect of these were able to significantly improve the response of the glioblastoma cells toward RT (155).

Cytoprotective autophagy induction by IR is largely contributed by ROS and ER stress determining the radiotherapy outcomes. The elevated ROS in irradiated cells generate oxidative damages to DNA, protein, and lipid causing ER stress and unfolded-protein response which in turn stimulate autophagy to eliminate the damaged cellular macromolecules. Attempts has continuously been made to develop a combination therapy involving autophagy inhibitors and IR to enhance radio-sensitization of tumors through induction of apoptotic cell death. Combining IR with autophagy inhibitors 3-MA or bafilomycinA1 (BafA1) restricts cell growth and proliferation whereas adding autophagy inducer rapamycin in the IR treatment regimen has induced cell proliferation, clearly demonstrating the differential response of the irradiated cells to autophagy modifiers. Similarly, in *in vivo* studies in whole-body irradiated mice models, rapamycin increased survival rates whereas chloroquine (CQ), an autophagy inhibitor, has lowered the survivability of the animals (136). HT29 colorectal cells which were p53 deficient have been radiosensitized after CQ treatment and autophagy inhibition (148). Interestingly, when combined with the mTOR inhibitor temsirolimus, CQ induced radiosensitization and apoptotic cell death in colorectal cancer cell lines (149). IR-induced activation of mTOR signaling was blocked by temsirolimus with autophagy induction whereas CQ inhibited the autophagy as evidenced by p62 and LC3-II expression levels. When combined, both mTOR signaling and autophagy were suppressed with concomitant induction of apoptosis in the IR-exposed cells. In a similar approach, everolimus and other PI3K/mTOR inhibitors in combination with CQ have shown increased anticancer effect where inhibition of mTOR downstream signaling accompanied by CQ mediated autophagy inhibition induces apoptosis in neuroendocrine tumor cell line BON1 (150). At low cytotoxic dosages, CQ was found to radio-sensitize bladder cancer cells, both *in vitro* and xenografted mouse models (165). CQ blocks the IR-induced DNA damage repair and activated apoptosis in the irradiated tumor cells by inhibiting autophagy. Autophagy inhibition by chloroquine enhances the radio-sensitivity of the cells with concomitant apoptosis induction associated with G1/G0 cell cycle arrest and reduction in cancer-initiating cell populations (166). Hydroxychloroquine (HCQ)-loaded mesoporous silica nanoparticles with enhanced cellular permeability and intracellular accumulation resulted in autophagy (cytoprotective) inhibition and a marked increase in IR-induced cell death in HCT116 colon cancer cells (167). Tumor xenograft mice models exhibit better tumor targeting of the HCQ-loaded nanoparticles. 3-MA which is a potent inhibitor of PI3K signaling has been found to inhibit radiation-induced autophagy and sensitize the esophageal cancer cells to IR with increased apoptosis and slower cell growth (152, 153). Moreover, the synergistic effect was observed in mice xenograft models with regression of tumor volume and reduction in the vasculature.

The available pre-clinical reports with both the autophagy activators and inhibitors indicated that combining these autophagy modifiers with IR has immense potential in avoiding radioresistance as well as in aggravating cytotoxic effects. However, due to the double edged effect of autophagy on the cancer cells, the effect of autophagic modulators on their survival becomes the function of the disease progression. Hence, while formulating a treatment regimen for clinical studies involving autophagic modulators, caution must be taken and information regarding the site and stage of the tumor mass, along with its genetic profile should be carefully considered.

## 6 Autophagy and radiation response of tumors: Clinical studies

Prompted by the compelling evidence from preclinical studies that suggested a role for autophagy in the radiation response of tumor cells and the effects of various modifiers of autophagy on the radiation response of tumors, clinical trials were initiated nearly two decades ago to validate these findings in different human malignancies. These studies have focused on the correlation between various regulators of autophagy and the response of tumors to radiotherapy and chemoradiotherapy, as well as the effects of different modifiers of autophagy on the response to radiotherapy and chemoradiotherapy. Although Pevonedistat, a NEDD8 Activating Enzyme inhibitor, and activator of protective autophagy has been extensively investigated either as a monotherapeutic or as part of a combined modality with chemotherapeutic drugs and immune modifiers (168), it has not been investigated so far in combination with radiotherapy.

### 6.1 Regulators of autophagy and tumor response to various therapies

The influence of various regulators of autophagy on the response of the tumor towards RT and CRT (Chemo-radiotherapy) evaluated in some of the human malignancies has shown an inverse relationship between the levels of these regulators and clinical response to RT or CRT. In nasopharyngeal carcinoma a high Beclin1 level correlated with poor response to CRT (169, 170). Similarly, elevated levels of ATG4B and LC3B were associated with poor response to the standard of care (RT and TMZ) in glioblastoma (171) and in prostate cancers, with high LC3A and low LAMP2 levels, were found to be resistant to RT (172). **Table 2** summarizes the outcome of clinical studies that investigated the relationship between the different autophagy regulators and tumor response toward RT or CRT.

**Table 2:** Clinical studies examining the relationship between regulators of autophagy and tumor response to radiotherapy and chemoradiotherapy.

Autophagy regulator	Tumors	Therapy	Findings	References
Beclin1	Nasopharyngeal carcinoma	Floxuridine + carboplatin and RT	High Beclin1 expression correlated with poor overall, progression-free, and distant metastasis-free survival	(147)
pATG4B and LC3B	Glioblastoma multiforme	TMZ and RT	Survival inversely correlated with pATG4B and LC3B	(173)
High LC3A/low LAMP2A	Prostate cancer	RT	Associated with resistance against RT	(170)

### 6.2 Targeting autophagy for improving the RT of tumors

Many small molecules, subdivided into seven different functional groups and targeting different regulators of autophagy, have been considered as potential adjuvants to RT and chemotherapy of cancer (174, 175). Of these, repurposing of the drug chloroquine (CQ) and its derivative hydroxyl chloroquine (HCQ), originally approved for the treatment of malaria and are known to disrupt the autophagosome formation, have been extensively investigated as mono-therapeutic as well as an adjuvant to radio and chemotherapies (176), although limited clinical trials have evaluated the efficacy of the other classes of autophagy targeting drugs.

A double-blind placebo-controlled trial with (CQ as an adjuvant to chemo-radiotherapy (RT+TMZ) demonstrated a significant improvement in the median survival of patients with glioblastoma as compared to the control (RT+TMZ) arm (177). Likewise, CQ was found to enhance the response of brain metastasis to whole-brain irradiation, without significant toxicity (178). Unfortunately, a Phase I/II clinical trial in stage IV small cell lung cancer evaluating the efficacy of a combination of CRT and CQ had to be terminated due to poor accrual (179).

Due to its lesser toxicity level the CQ derivative HCQ (180), has been extensively investigated in clinical trials both as a mono-therapeutic as well as in combination with chemo- and radiotherapy. Although inhibition of autophagy is clinically feasible with HCQ and also enhances the efficacy of chemo- and radiotherapy of many human malignancies, dose-limiting toxicity, mainly in the form of retinopathy has limited the efficacy and its utility as an adjuvant to radiotherapy of tumors (181, 182). A Phase II clinical trial was initiated in 2007 in pancreatic cancer (NCT01494155) that evaluated the toxicity and efficacy of a combination of short course chemo-radiotherapy (SCRT; gemcitabine and photon/proton RT) and HCQ. However, a long-term follow-up has revealed that although the combined treatment of HCQ and SCRT was well tolerated, significant improvement in terms of survival benefit was not observed (183). Unfortunately, till date, no information is available in the public domain regarding the outcome or status of many clinical trials initiated in recent years to evaluate the feasibility, toxicity, and efficacy of combining CQ or HCQ with radiotherapy or chemoradiotherapy for the treatment of different solid tumors.

The mTOR inhibitors Temsirolimus and Everolimus are known to inhibit the initial events of autophagy. No significant improvement in terms of patient survival was observed when glioblastoma patients were treated with a therapeutic regime combining Temsirolimus and Everolimus with chemoradiotherapy (184, 185). Interestingly, Nelfinavir; a PI3K/Akt inhibitor has been found to provide moderate survival benefits without severe grade 3/4 toxicity in LAPC (Locally advanced pancreatic cancer) and NSCLC (186–189) patients.

An overview of the clinical trials that target the autophagy machinery is presented in **Table 3**. Through the course of these trials many limitations of the existing autophagy targeting drugs have been identified that compromise the efficacy of the therapeutic regimen that involves them. Toxicity, attenuated efficacy in the acidic milieu of TME, and inability to reliably monitor the autophagic flux are among a few. Since most, if not all, modifiers of autophagy investigated clinically so far do not exclusively alter autophagy, but affect other signaling pathways of radiation response, it is reasonable to expect heterogeneity in the response of tumors to a combined regimen of RT and autophagy targeting drugs. Since most of the autophagy regulating genes have moonlighting properties where they have other functions, the therapeutic benefit of combined therapies involving autophagy modifiers may be obscured by their effects on other targets. Thus, therapies combining autophagy targeting drugs with radiation and/or chemotherapeutic agents have not elicited encouraging response either due to the lack of proper selection of patients (which should have been done based on a complete understanding of the biological behavior of the tumor) and/or our inability to adopt the best approach for manipulating autophagy in individual patients. This is particularly relevant when combining RT with modifiers of autophagy as radiation-induced autophagy can be either pro-survival or promote death in a context-dependent manner (49). This limitation can be overcome to a very large extent by complete characterization of tumors for their biological behavior particularly related to the status of regulators of various signaling pathways triggered by radiation damage, especially the status of the autophagy regulators.

**Table 3:** Overview of the clinical trials targeting autophagy for improving radiotherapy of tumors.

Autophagy targeting drugs	Tumors	Trial	Therapy		Findings	References
Autophagosome (formation) Inhibitor						
Hydroxychloroquine (HCQ)	Glioblastoma multiforme	I/II	Conventional RT with TMZ	Dose-limiting toxicity and no significant improvement in survival		(181) NCT02738582
	Pancreatic cancer	I/II	CRT with Photon or Proton therapy	Well tolerated, but no significant survival benefit		(180) NCT01494155
Chloroquine (CQ)	Recurrent glioblastoma	I/II	Conventional RT with TMZ	Feasibility established		(176)
	Glioblastoma multiforme	III	Conventional RT with TMZ	Improvement in survival and reduced death rate		(177) NCT00224978
	Brain metastasis	II	Whole-brain irradiation	Enhanced tumor response without toxicity		(178)
	Stage IV Small Cell Lung Cancer	I/II	Chemoradiotherapy	Terminated due to poor accrual		(179) NCT01575782
mTOR inhibitors						
Temsirolimus	Recurrent glioblastoma	I/II	Conventional RT with TMZ	Clinical benefit in 335 patients		(184)
Everolimus		II		No significant survival benefit		(185)
PI3/Akt inhibitors						
Nelfinavir (HIV protease inhibitor)	Locally advanced pancreatic cancer	I/II	Chemoradiotherapy	Moderately improved tumor response, but Grade 3 & 4 GI toxicity		(186)
		I/II	Stereotactic body radiotherapy (SBRT)	MTD identified		(187)
	Non-small cell lung cancer	I/II	Chemoradiotherapy	Median survival of 12 months and progression-free survival of 41 months, without grade 3/4 toxicity		(188, 189)

The NCI identifier numbers of the clinical trials are mentioned along with reference to the literature.

## 7 Summary

Current understanding implicates autophagy in several cellular events including biological responses to stress caused by ionizing radiation and a variety of other therapeutic agents. Autophagy appears to be largely pro-survival while promoting death under certain circumstances in a context-dependent manner. Its emerging role in tissue, as well as its effects in systemic level following focal irradiation of tumors, suggests its ubiquitous impact in therapeutic responses to RT, which has prompted several clinical studies to target autophagy for improving the efficacy of therapy. However, encouraging clinical responses have yet not emerged from the Phase I/II of the clinical trials conducted so far which, unfortunately, has precluded a therapeutic regime consisting of an autophagy modifies as the principal component or adjuvant to RT/CRT to proceed towards the Phase III of clinical trials.

## Author contributions

AR, SB, and BD worked in conceptualizing, designing, and preparing the manuscript. All authors contributed to the article and approved the submitted version.

## Acknowledgments

The authors wish to thank Dr. Madhuri Chaurasia, Weizmann Institute of Science, Rehovot, Israel for the helpful discussions in preparing the manuscript.

## Conflict of interest

The authors declare that the research was conducted in the absence of any commercial or financial relationships that could be construed as a potential conflict of interest.

## Publisher’s note

All claims expressed in this article are solely those of the authors and do not necessarily represent those of their affiliated organizations, or those of the publisher, the editors and the reviewers. Any product that may be evaluated in this article, or claim that may be made by its manufacturer, is not guaranteed or endorsed by the publisher.

## Abbreviations

ATG, Autophagy; ATM, Ataxia,telangiectasia; BRCA, Breast cancer gene; cGAS, Cyclic GMP AMP synthase; CHK1 & 2, Checkpoint kinase 1 & 2; CDK – Cyclin,dependent kinase; CSC – Cancer stem cells; DAMP, Damage associated molecular patterns; DDR, DNA damage repair; DSB, Double, strand breaks; EMT, Epithelial mesenchyme transformation; HIF, 1 – Hypoxia, inducible factor 1; HR, Homologous recombination; IR, Ionizing radiation; LET, Linear Energy Transfer; NHEJ , Nonhomologous end, joining; NTE, Non, target effects; RIBE, Radiation,induced bystander effects; RT, Radiotherapy; SBRT – Stereotactic body radiotherapy; STING, Stimulator of interferon genes; TGF, Transforming growth factor; TME, Tumor microenvironment; VEGF, Vascular endothelial growth factor.
